# Dynamic Allostery of the Catabolite Activator Protein Revealed by Interatomic Forces

**DOI:** 10.1371/journal.pcbi.1004358

**Published:** 2015-08-05

**Authors:** Maxime Louet, Christian Seifert, Ulf Hensen, Frauke Gräter

**Affiliations:** 1 Heidelberg Institutes for Theoretical Studies gGmbH, Heidelberg, Germany; 2 Eidgenössische Technische Hochschule Zürich, Department of Biosystem Science and Engineering, Basel, Switzerland; 3 CAS-MPG Partner Institute and Key Laboratory for Computational Biology, Shanghai, China; University of Wisconsin-Madison, UNITED STATES

## Abstract

The Catabolite Activator Protein (CAP) is a showcase example for entropic allostery. For full activation and DNA binding, the homodimeric protein requires the binding of two cyclic AMP (cAMP) molecules in an anti-cooperative manner, the source of which appears to be largely of entropic nature according to previous experimental studies. We here study at atomic detail the allosteric regulation of CAP with Molecular dynamics (MD) simulations. We recover the experimentally observed entropic penalty for the second cAMP binding event with our recently developed force covariance entropy estimator and reveal allosteric communication pathways with Force Distribution Analyses (FDA). Our observations show that CAP binding results in characteristic changes in the interaction pathways connecting the two cAMP allosteric binding sites with each other, as well as with the DNA binding domains. We identified crucial relays in the mostly symmetric allosteric activation network, and suggest point mutants to test this mechanism. Our study suggests inter-residue forces, as opposed to coordinates, as a highly sensitive measure for structural adaptations that, even though minute, can very effectively propagate allosteric signals.

## Author Summary

The Catabolite Activator Protein (CAP) is a well-studied example for how cellular catabolite levels are integrated into the gene regulation. Its affinity for a specific stretch of DNA can be switched on by the binding of two nucleotide molecules termed cAMP to its two protomers. Even though the nucleotides occupy structurally identical binding pockets, the second cAMP binding occurs at an affinity orders of magnitude lower than the first cAMP binding. The question arises how, in the absence of structural changes, the first binding can affect the second. An answer from experiments has been that the communication is largely of entropic nature, i.e. the second cAMP binding would lead to a pronounced reduction in atomic fluctuations of the protein without affecting the atomic mean positions. We here revisited this question by performing Molecular Dynamics simulations. By measuring correlations of forces, a newly derived method outperforming the more common coordinate-based approach, we could recover the previously determined entropic penalty. In addition, however, we observed unobtrusive structural changes of side-chain interactions leading to the occlusion of the second binding pocket that add a critical ‘enthalpic’ component hitherto overlooked. Our study provides a mechanistic view onto the intriguing anti-cooperativity of CAP.

## Introduction

**Fig 1:**
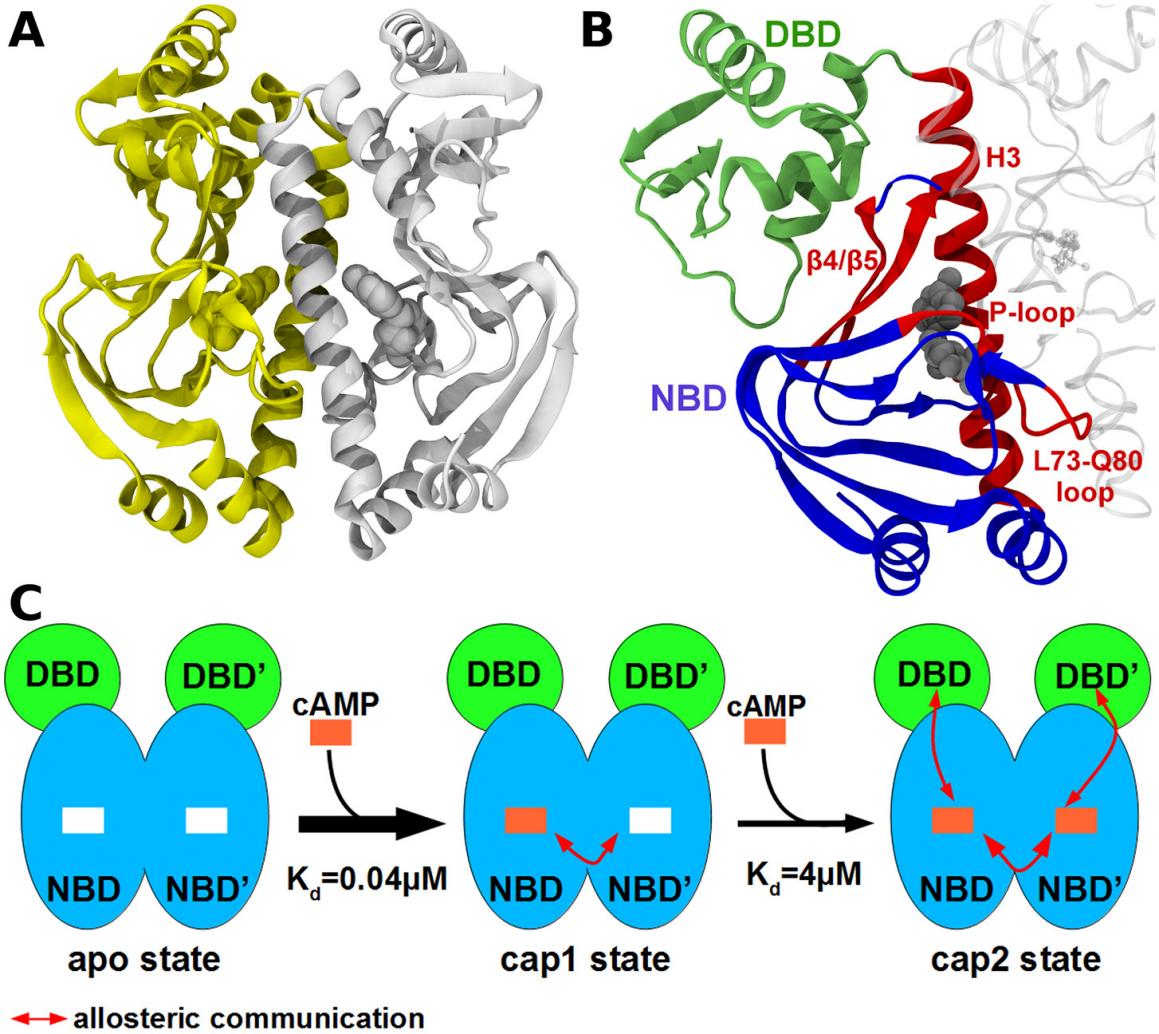
Overview of the CAP structure and allostery. (A) CAP homo-dimer in the two-liganded state known from X-ray diffraction (PDB id: 1G6N [9]). The first protomer is depicted in white cartoon and the second is in yellow cartoon, the two cAMP are represented as balls. (B) Representation of the important region of CAP, the DNA Binding Domain (DBD) is in green, the Nucleotide Binding Domain (NBD) in blue and hot-spots delineated by FDA are in red, the cAMP is in grey balls. (C) Schematic representation of cAMP binding to CAP and allosteric communications between the two protomers. The DBD is shown in green, the NBD is shown in blue. The first cAMP (orange square) binds with a high affinity, whereas the second cAMP binds with a lower affinity (negative cooperativity). The cAMP binding provokes conformational changes in the DBD allowing DNA binding.

The regulation of DNA transcription is one of the key factors for the control of cell functions, a classic example of which is the lac-operon model. In this gene regulatory mechanism, the catabolite activator protein (CAP) promotes DNA transcription initiation. The homodimeric protein CAP (Fig 1A) binds cyclic adenosine monophosphate (cAMP) and DNA. The N-terminal nucleotide binding domain (NBD; Fig 1B blue) acts as a dimerization domain and is globular. The C-terminal DNA binding domain (DBD; Fig 1B green) is bound via a flexible linker to the cAMP binding domain and consists of a helix-turn-helix motif. CAP activity is regulated mainly through negative cooperativity of the two cAMP binding events: the binding affinity of the second cAMP is reduced by nearly two orders of magnitude [1–3]. Once CAP is activated by cAMP, it binds to a specific DNA region, and thereby enhances downstream transcription [4–6]. While the binding of CAP to DNA is largely understood [7], the allosteric mechanism of cAMP binding is still elusive. The puzzling fact that the binding of the first cAMP leads to no recognizable structural change at the second binding pocket [8] but still reduces its binding affinity by two orders of magnitude [1–3] has led to numerous investigations. The crystal structure of singly cAMP bound CAP shows a distance of more than 20 Å between the two binding pockets [9], and therefore direct Coulombic interaction between the two cAMP cannot explain the negative cooperativity. Also, CAP structures, resolved by X-ray or Nuclear Magnetic Resonance (NMR), with no or two cAMP molecules bound, do not explain the observed allostery. Instead, NMR measurements and isothermal calorimetry (ITC) on a truncated construct (no DBD) [8] revealed that the binding of cAMP gives rise to a primarily dynamics-driven allosteric mechanism. More specifically, a change in dynamic fluctuations in the unliganded protomer upon cAMP binding to the other protomer render the binding of a second cAMP molecule entropically unfavorable. Similarly, changes in the conformational entropy of CAP due to a global redistribution of internal dynamics was shown using NMR and ITC to also decisively impact DNA binding. Molecular Dynamics simulations and free energy calculations have supported this view on CAP dynamic allostery [10].

These and similar results of other allosteric proteins have led to an ensemble-based view of protein allostery [11,12], according to which local or global conformational motions on various time scales are affected by ligand binding [8,13], thereby triggering a change in protein affinity or activity. Nevertheless, even in the absence of conformational changes, allostery requires the distant effector and functional sites of a protein such as CAP to be coupled. Thus, we here propose a well-defined communication pathway, i.e. a subset of residues primarily involved in correlating effector binding with protein activity, as the basis of the dynamic allostery of FAK [14].

We here aim at determining the distinct allosteric pathways that could explain the negative cooperativity of cAMP binding to CAP and its impact on CAP-DNA association. To this end, we used Force Distribution Analysis (FDA) [15,16], a Molecular Dynamics (MD) based analysis of inter-atomic forces propagating through the structure upon an external perturbation, which here is cAMP binding. This method is similar to calculations of molecular stresses [17,18]. FDA has previously allowed to track pathways underlying structural allostery [19,20] as well as dynamic allostery as observed in the methionine repressor MetJ, another gene regulatory protein [21].

We compared inter-atomic forces within three different forms of CAP: the apo, the single and double cAMP bound forms, which we are going to refer to in the following as apo, cap1 and cap2, respectively. FDA allows to highlight atomic interactions involved in allosteric signal transmission even for very small atomic displacements, in contrast to other computational methods which typically rely on large-amplitude motions. In particular, even if atomic displacements are largely absent, and allostery instead is largely based on fluctuations redistributing within the protein upon effector binding, changes in mean inter-atomic forces capture these dynamic effects due to the anharmonic nature of the underlying potential [21]. We thoroughly validate the MD simulations of the three states of CAP by comparison to experimental X-ray and NMR data. We detect a primary signalling pathway between the two cAMP binding site of the CAP homo-dimer similarly involved in both cAMP binding events, which can explain the anti-cooperativity of cAMP binding. Also, we put forward a symmetric pathway between the NBD and DBD of CAP critically involved in signalling towards the DNA binding site. Our results highlight hot spots of CAP’s dynamic allostery, which are testable by experiments, and suggest that allosteric signalling pathways and entropy driven allostery do not exclude each other but instead can represent different perspectives of the same mechanism.

## Results

### Intrinsic Dynamics of CAP

In the following, we will refer to the apo state, the single and double cAMP bound states of CAP as apo, cap1 and cap2. Starting from the high-resolution crystal structure of cap2 (PDB id: 1G6N [9]), we performed 9 independent Molecular Dynamics (MD) simulations of each of the three differently liganded states (see Material and Methods section). The production time of the 27 simulations was 100ns, resulting in 2.7μs of CAP trajectories.

We validated the accuracy of the simulations by comparing atomic fluctuations with X-ray (B-factor) and NMR (Squared Generalized Order Parameters for the Methyl Group Symmetry Axis, S^2^axis) data as well as biologically relevant motions with available experimental data. The calculated residual root mean square fluctuation (RMSF) of cap2 correlates reasonably (R = 0.84) with the experimental B-factors of the X-ray structure (PDB id: 1G6N, S1 Fig). Both the crystal structure and the cap2 simulations show (S1 Fig) high fluctuations only in the outer loops of the DNA-binding domain (DBD). Interestingly, the B-factors and the RMSF indicate higher fluctuations for one protomer than the other, an asymmetry that is not reflected in the NMR measurements, for which signals inherently represent averages over the two protomers.

To compare our simulations even more directly with available NMR data, we additionally calculated CH3 order parameters (S^2^_axis_) for cap2 as described by Hu et al. [22] (see Material and Methods section). We obtained an average order parameter of 0.511, which is in satisfying agreement with the average value of 0.529 from NMR experiments [23], and follows nicely the trend between MD and NMR based average order parameters observed previously for other proteins [24]. Interestingly, the 2-cAMP bound CAP falls into the more flexible regime of the seven other proteins compared therein. A residue-to-residue comparison (S2 Fig) yielded a correlation of 0.61 between MD and NMR data for cap2 (whole protein), and of 0.71 considering only the NBD. Correlation between S^2^axis derived from MD and NMR has been previously found to be similarly low for other proteins [24]. In our case, it might be particularly challenged by starting the MD simulations from an X-ray structure (pdb id: 1G6N) instead of a cAMP-bound solvent NMR structure, for which no wild-type homo-dimer structure with 2 cAMP has yet been solved. Overall, with regard to entropies and order parameters, we find our MD simulations to largely reproduce the cAMP-dependent flexibility of CAP.

We next analysed the similarity of the collective motions observed in our MD simulations with those described by the corresponding experimental structures. We computed collective modes of motion for the three different states from each set of 9 simulations, and compared them to the ‘inactivation motion’ as described by the apo and cap2 NMR structures (2WC2 and 1G6N, respectively [9,25]). Interestingly, the 4th eigenvector, obtained from the apo simulations which is expected to relax towards the apo state, indeed correlates with the experimentally suggested inactivation motion (S1 Movie) with a correlation coefficient of up to 0.54 (or 0.61 for 4th eigenvector of cap1). By contrast, a lower correlation coefficient, of maximum 0.42, was found between the 20 largest amplitude eigenvectors of the 2cAMP bound state and the experimental inactivation motion. This indicates that the computed dynamics partially and yet specifically capture the functional motions described by experimental structures.

**Fig 2:**
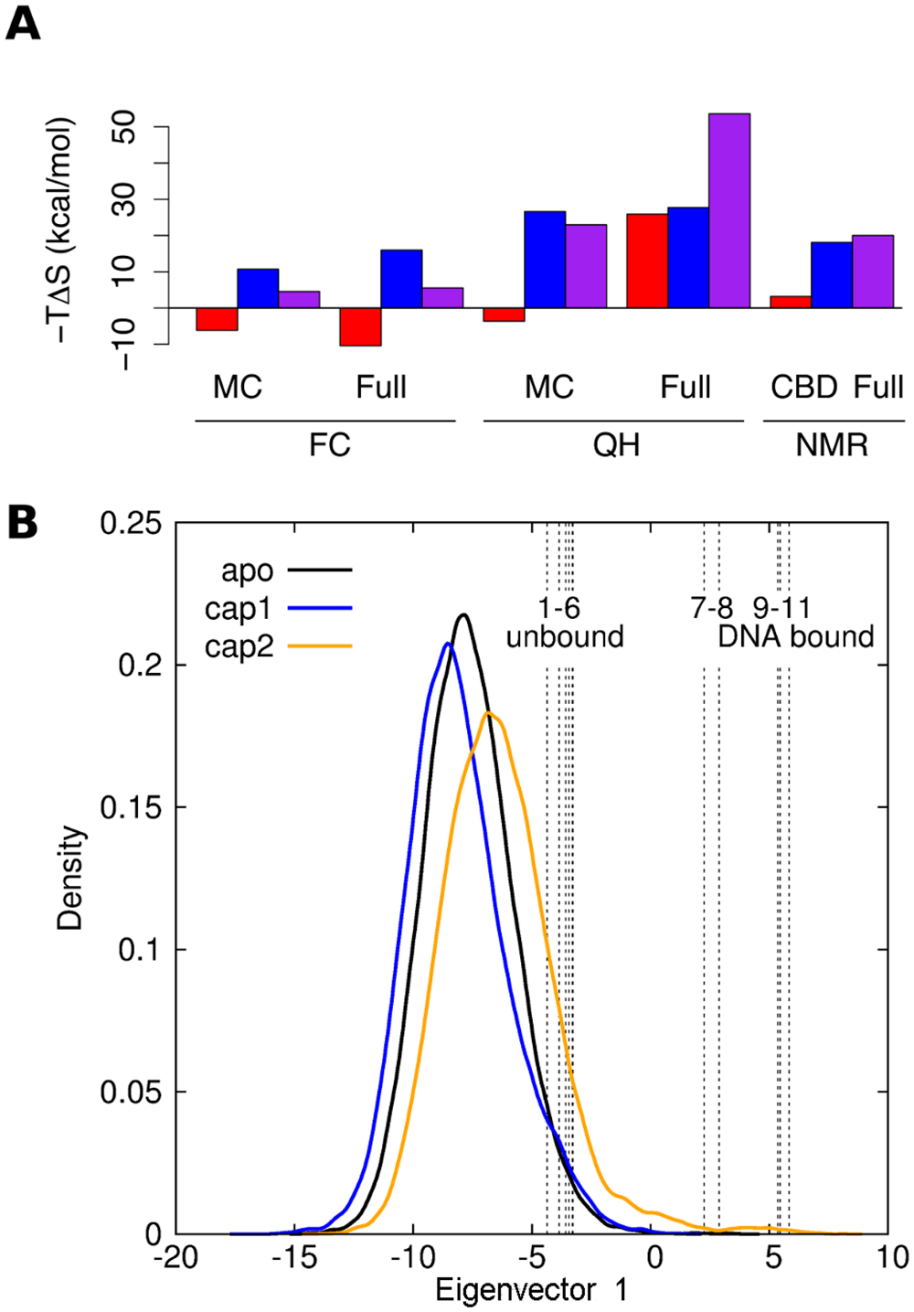
Global motions of CAP. (A) Estimated entropic contributions-TΔS to the binding free energy of the first (red) or the second (blue) cAMP binding event, and the overall entropy change for the binding of both cAMP (purple). Estimates from force covariance (FC) and quasi-harmonic (QH) analyses of either protein main chain (“MC”) or the full protein including DBD (“full”). For comparison, NMR-based estimates (“NMR”) are given for the entropy change of the first and second binding event of a truncated CAP construct (“CBD”) without DBD [8] and, respectively, for the binding of both cAMP to the full protein (“full”) [23]. (B) Functional motion of CAP for DNA binding as sampled in MD simulations. Projection of CAP X-ray structures and all simulation data from apo (black), cap1 (blue) and cap2 (orange) states on the first eigenvector obtained from a PCA of available 2 cAMP-bound X-ray structures, either solved in absence of DNA (1–6: 1GN6 [9], 1HW5 [27], and 1I6X, 3RDI, 3ROU, 1I5Z –all unpublished) or in presence of DNA (9–11: 1RUO [28], 1RUN [28] and 1CGP [26]). The two intermediate structures (PDB ids: 3QOP (unpublished) and 3KCC [29]) are not bound to DNA but to two cAMP molecules, localized in between the DBD and β-strand 5, triggering a rotation of the DBD.

As a final sanity check of our models, we also compared the experimentally and computationally observed motions of the DBD involved in DNA binding. To this end, we projected our simulations on the first eigenvector derived from 11 X-ray structures, all of which possessed two cAMP, but for which the DNA could be absent or present. This eigenvector corresponds to the previously described large-scale DNA binding motion [9,26], and involves an almost rigid rotation of the DBD by ~25 degrees. X-ray structures with bound DNA clearly separated from structures not coupled to DNA along this eigenvector, with only two intermediate conformations (which show non-canonical cAMP binding). Remarkably, in spite of the limited time scale of our MD simulations, projections of conformations explored during the MD simulations and experimentally resolved structures [9,26–29], on this particular eigenvector showed that the cap2 state is able to follow this motion further than the apo and cap1 states and even overlaps with DNA-bound X-ray structures (Fig 2B). This suggests that even though we started from the same active cap2 structure, the sampled conformational space partially diverged during the unbiased MD simulations along directions in agreement with experiments. We note that even, the cap2 ensemble preferentially populates a region to the left of the experimental structures, a diversion which however, is minor, and could be due to crystal packing effects.

### Configurational Entropy Estimations

Because it has been shown experimentally that the binding anticooperativity in CAP is entropy driven [8,23], we compared experimental and simulated entropy changes upon cAMP binding (Fig 2A). Configurational entropies were obtained from both our recently developed force covariance (FC) estimator [30] and the more established quasi-harmonic (QH) approximation [31] for the full protein. Briefly, QH and FC entropy estimators both assume the system to be harmonic and estimate the vibration frequencies from correlated atomic coordinates (QH) or from correlated atomic forces (FC). Atomic forces have been shown to deviate less from harmonics than coordinates, such that the harmonic approximation becomes more accurate if based on force covariance [30].

An entropic penalty for the overall process of −TΔ_Scap2-apo_ = 20 kcal.mol^-1^ due to the binding of two cAMP ligands has been measured for the full protein construct with NMR, through S_2axis_ order parameters [23]. This result is in qualitative agreement with our simulations, which corroborate an estimated overall entropic penalty ranging from 6 to 54 kcal mol^-1^, depending on the selection of atoms for the analysis as well as the estimation method used (Fig 2A, magenta).

Available experimental NMR NH-bond order parameters of a truncated construct (without DBD) suggested a small entropic penalty for the first cAMP binding of −TΔS_cap1-apo_ = 3.2 kcal.mol^-1^, followed by a much larger penalty of −TΔS_cap2-cap1_ = 18.1 kcal.mol^-1^ for the second cAMP binding event [8]. From our simulations, both methods (QH and FC) instead consistently estimate the entropy changes of the first binding event −TΔS_cap1-apo_ to be small but favourable (−10 vs −4 kcal mol^-1^ for FC and QH, respectively). QH, however, is very sensitive to the selection of atoms included in the analysis, and implausibly estimates an entropy change of 25 kcal.mol^-1^ for the full protein. For the second binding event, QH and FC both corroborate the experimentally measured entropic penalty, ranging from 10 to 26 kcal mol^-1^ depending on the selection of atoms for the analysis. The partial discrepancy for the first binding event may partly be attributed to the fact that the NMR experiments used a truncated construct without DBD. We hypothesize that full length CAP in contrast to the truncated protein shows a favourable entropy change upon binding the first cAMP, which is yet to be tested experimentally.

### Force Distribution Analysis

Having validated the set of MD simulations comprising the apo, cap1, and cap2 states, we next asked how cAMP binding is allosterically regulated such that it occurs anticooperatively. To reveal the allosteric network of cAMP binding in CAP, we calculated the change in pairwise residue forces upon each nucleotide binding event, averaged over the nine independent 100 ns simulations. We observed convergence of the force differences after approximately six of the nine MD simulations (S3 Fig).

### First Binding Event

By convention, the first protomer will refer to the one where a cAMP molecule is present in the cap1 state. In order to distinguish amino acids from both protomers, we added a prime to residue numbers of the second protomer, such as Arg123’.

**Fig 3:**
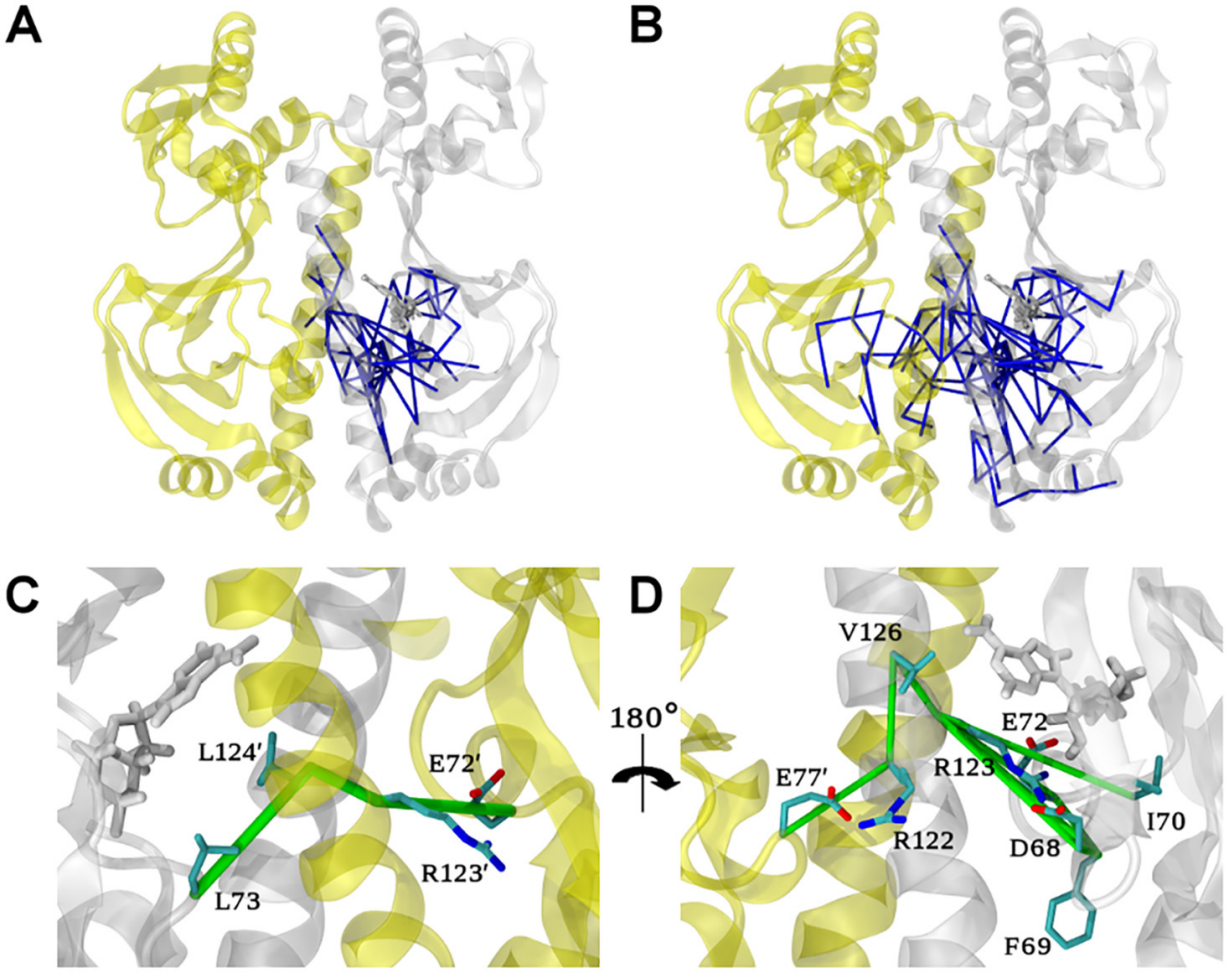
Allosteric network upon binding the first cAMP obtained from FDA. Residue pairwise forces difference between the apo and the cap1 states are shown as blue sticks at a 50 pN (A) and 40 pN cut-off (B) for the CAP homo-dimer represented as cartoon. The first and second protomers are depicted in white and yellow, respectively. The cAMP molecule (white) and key residues are represented as sticks. (C, D) Zoom of (B) to highlight two distinct allosteric connection pathways termed A (C) and B (D) in the allosteric network between the two protomers at a cut-off value of 40 pN. Note that D is rotated by 180 degrees with respect to C.

To explore the perturbation and potential allosteric communication caused by the first binding event, we first analysed the network of force difference between the apo and the 1cAMP bound (cap1) states. Fig 3 shows a graphical representation of the changes of residue-residue forces upon binding the first cAMP, with edges drawn between residues that exhibit force differences beyond a given cut-off. Only the largest connected network (in terms of number of amino-acids involved) is shown to further reduce the noise due to the undersampling in the MD simulations.

At a force difference cut-off of 50 pN, the largest network is located around the site of perturbation, i.e. the binding site of the first cAMP (Fig 3A). This network involves mainly the first protomer, especially residues in direct contact with the nucleotide, but also residues His31 to Ala36 in contact with the P-loop (see Fig 1B) and residues from the loop Leu73-Gln80. Remarkably, the force network spans residues all the way down to the N-terminal half of the H3-helix (see Fig 1B) of the first protomer and five residues of the H3-helix of the second protomer (Ala122’, Arg123’, Leu124’, Gln125’ and Thr127’). We also observed signal propagation from Asp68 to the anti-parallel β4/β5-sheet (Ser46, Val49, Ser62, Tyr63, Leu64 and Asn65, Fig 1B).

A larger yet weaker force network becomes visible at a decreased cut-off of 40 pN (Fig 3B), where we observed signal propagation from the first to the second cAMP binding site. This network shows two distinct connection pathways, referred to in the following as pathways A and B, with a distinct set of molecular interactions at the protomer interface (Fig 3C and 3D).

**Fig 4:**
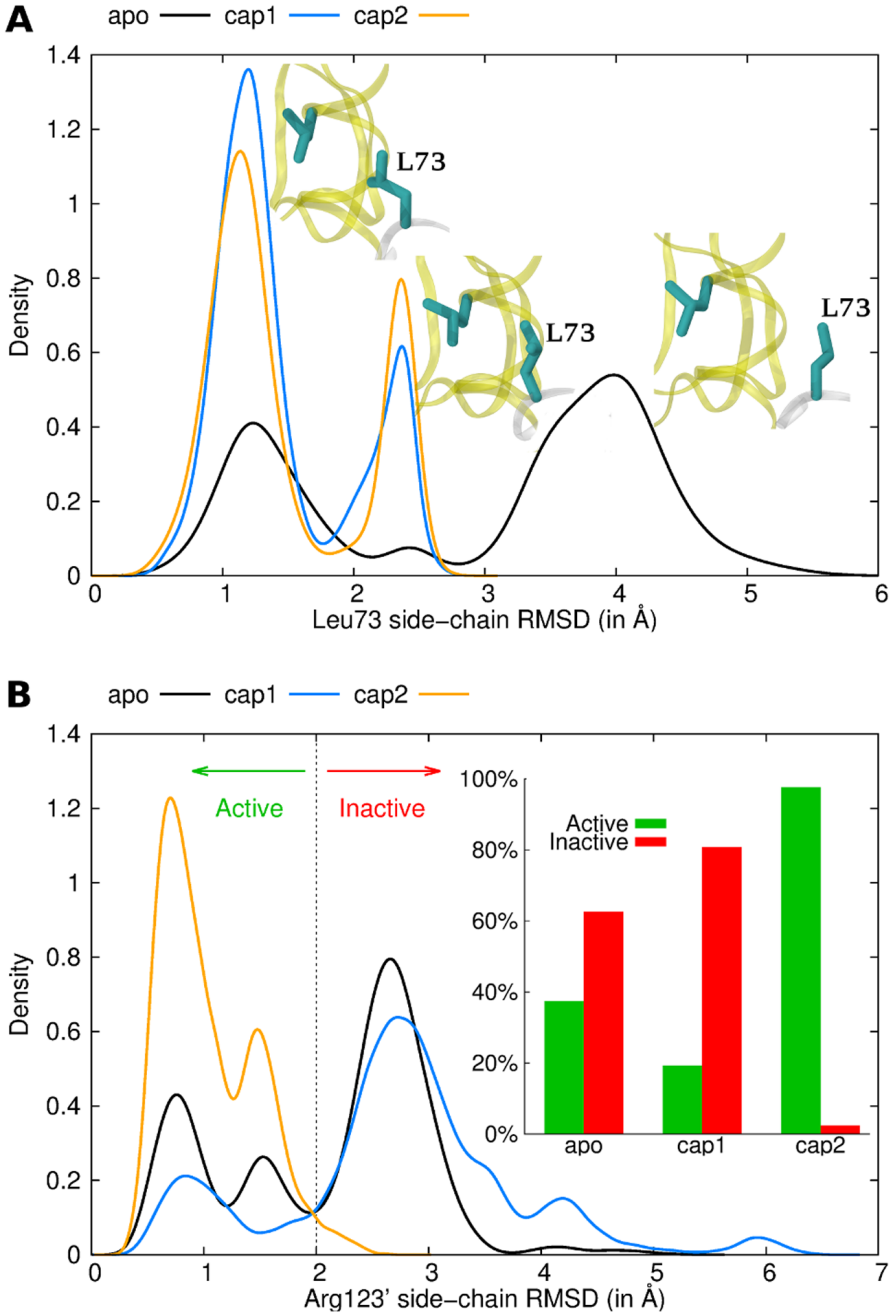
Conformational ensemble of Leu73 (A) and Arg123’ (B), involved in pathway A, for the apo (black), the cap1 (blue) and the cap2 (orange) states. The protein is represented as cartoon in white (first protomer) and yellow (second protomer). Key residues are represented as cyan sticks. Histograms of local RMSDs are shown. The average structure of all CAP simulations was used as reference structure for the fitting and RMSD calculation, allowing to directly compare conformations of different states. Only residues around a 6Å cut-off were used for the fitting to track local rearrangements without including global motions.

Pathway A is composed of only three specific residue pairs: Leu73-Leu124’, Leu124’-Arg123’ and Arg123’-Glu72’. The presence of the ligand in the binding pocket results in a stronger hydrophobic packing interaction between Leu73 and Leu124’ (Fig 4A). The signal is then propagating through backbone interactions from Leu124’ to Arg123’. Finally, the Arg123’ side-chain gains flexibility upon binding of the first cAMP (Fig 4B), leading to altered pairwise interactions between Arg123’ and Glu72’. Arg123’ is in close contact with the cAMP molecule in the second protomer, and mutation of this residue drastically decreases CAP activity [32]. Likewise, Glu72’ interacts directly with the second cAMP in the 2 cAMP-bound X-ray structure (the starting structure of our MD simulations) through a hydrogen bond with the sugar moiety. Also this residue has been shown to be crucial for nucleotide binding and CAP activity, as shown in site-directed mutagenesis studies [33]. Pathway A as revealed by FDA now suggests these two residues to be directly involved in the allosteric communication for anticooperative binding of cAMP.

Pathway B mainly involves Arg123 and the residue pair Arg122-Glu77’, both bridged by Val126 (Figs 3D and 4B). Nucleotide binding strongly modifies the Arg123 conformation. Indeed, the force difference network identified a number of residues with different interaction patterns with this arginine (including Asp68, Phe69, Ile70 and Glu72). This shift in Arg123 conformations triggers slight modifications in the arginine backbone that are transmitted to the Arg122 through the H3-helix. As a result, the Arg122 side-chain is more mobile and has weaker interactions with Glu77’. We observed even stronger repulsion in the cap1 state, as the non-bonded energy term is more positive (S4 Fig). Glu77’ shows stronger interactions with its surrounding residues, including Gln80’ through side-chain interactions (a hydrogen bond was observed over 13% of the apo trajectory against 34% in the cap1 state, S4 Fig). The modified polar interaction network of pathway B bridges the two promoters and thereby complements the first signal transduction (pathway A) between the nucleotide binding pockets. Interestingly, the observed structural adaptations upon the first cAMP binding event partially also involve dynamical changes of the same residues (S5 Fig). Most importantly, we observed stiffening, measured by residual dihedral order parameter differences, in the loop comprising residues 68–72 and the P-loop region of the first protomer as well as residues Glu72’/Glu77’ of the second protomer, all of which highlighted by FDA.

### Second Binding Event

**Fig 5:**
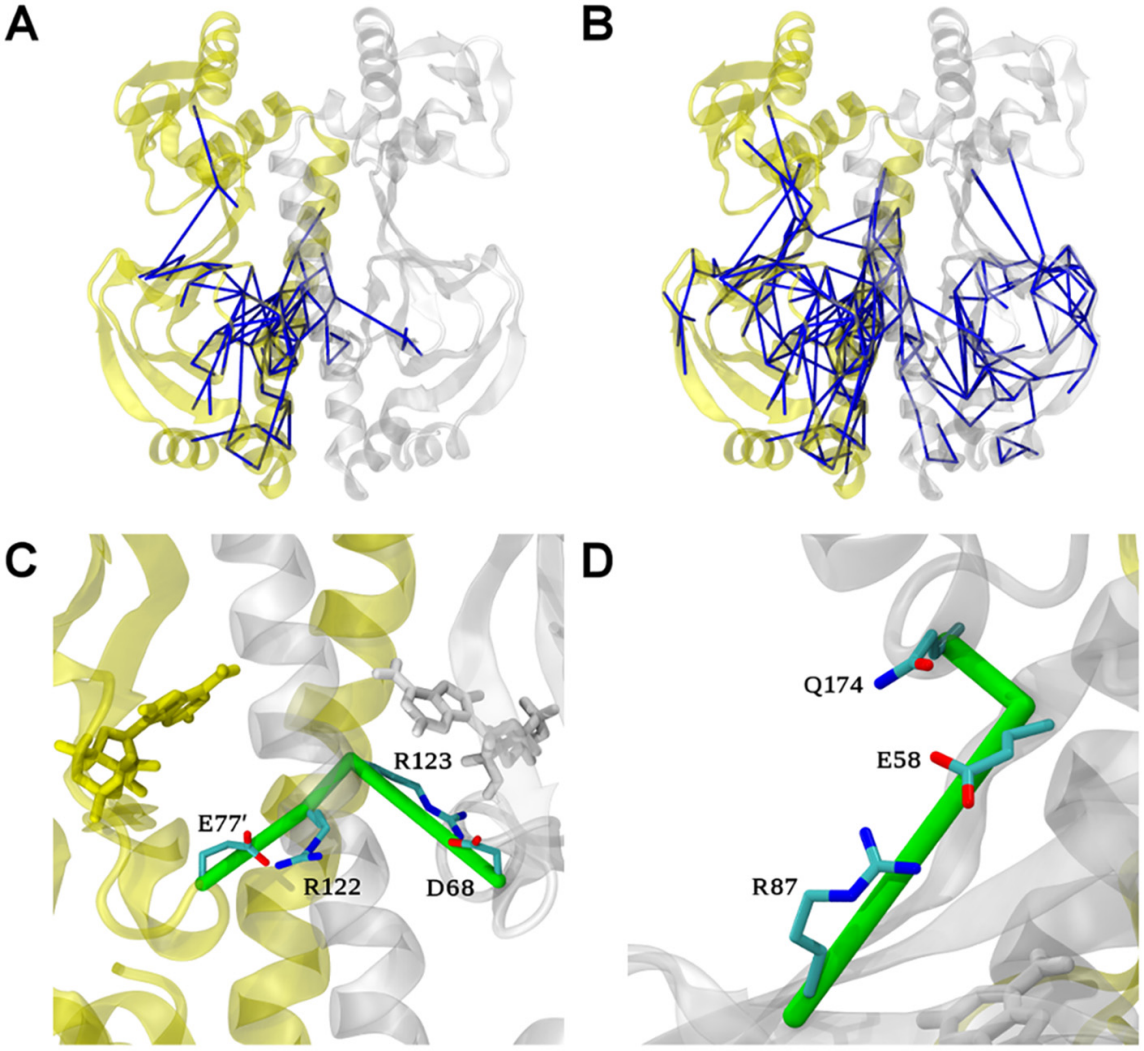
Allosteric network upon binding the second cAMP obtained from FDA. Residue pairwise force differences between the cap1 and cap2 states are shown as blue sticks at (A) 50 pN and (B) 40 pN cut-off. (C) Zoom of B highlighting the allosteric connection pathway between the two protomers, which resembles pathway B in Fig 3D. (D) Zoom of B, highlighting the force changes in the Glu58-Arg87 and Glu58-Q174 pairs which connects the NBD with the DBD and which are symmetrically present in both protomers.

Binding of the second cAMP entails local force changes within host protomer resembling those already seen for the first binding event (Fig 5A, also compare Fig 3A). Again, large residue-residue force differences were observed in the protomer hosting the additionally bound nucleotide, including the P-loop region and residues nearby (His31’ to Ala36’), the loop from Leu73’ to Gln80’ and also again the upper β4/β5-sheet (see Fig 1B). Similar interaction changes, upon the binding of the first and second cAMP, at least in the proximity of the binding pockets, are expected, given the high similarity of ligand-protein interactions. FDA encouragingly recovered this expected behaviour. However, at the same cut-off of 50 pN, the network after the second binding event now extends over ~15 Å from the rather local network around the cAMP binding cleft detected upon the first binding event. It now reaches the β-strands 2 and 7 to finally join the P-loop of both protomers, and even residues in close contact with and within the DBDs (Fig 5A and 5B). This new distant signal propagation towards the DBD is likely connected to the DBD activation motion observed in our MD simulations (Fig 2) and in available experimental structures [9,25]. The long-range nature of the cap1-cap2 force network is also in agreement with the global stiffening of CAP after the binding of the second cAMP observed experimentally [8]. An even larger, but slightly weaker (40 pN) force network now reaches all the way to the C-terminal region of the H3-helix (see Fig 1B). We would like to emphasize that this network was absent before binding of the second cAMP. This region is crucial for regulation and is known to undergo large conformational changes after CAP activation. Experimental structures showed that cAMP binding promotes addition of two helix turns at this C-terminal region [25,34].

FDA identifies a single connection pathway between the two protomers for the second nucleotide- binding event, involving almost the same residue pairs we observed for the allosteric propagation of the first binding event. (Fig 5C) Indeed, the previously described pathway B is very similar to the one involved in the signal transmission upon the second cAMP binding event, again including Glu77’-Arg122 and Arg123-Asp68, bridged by modified backbone interaction between Arg122 and Arg123.

**Fig 6:**
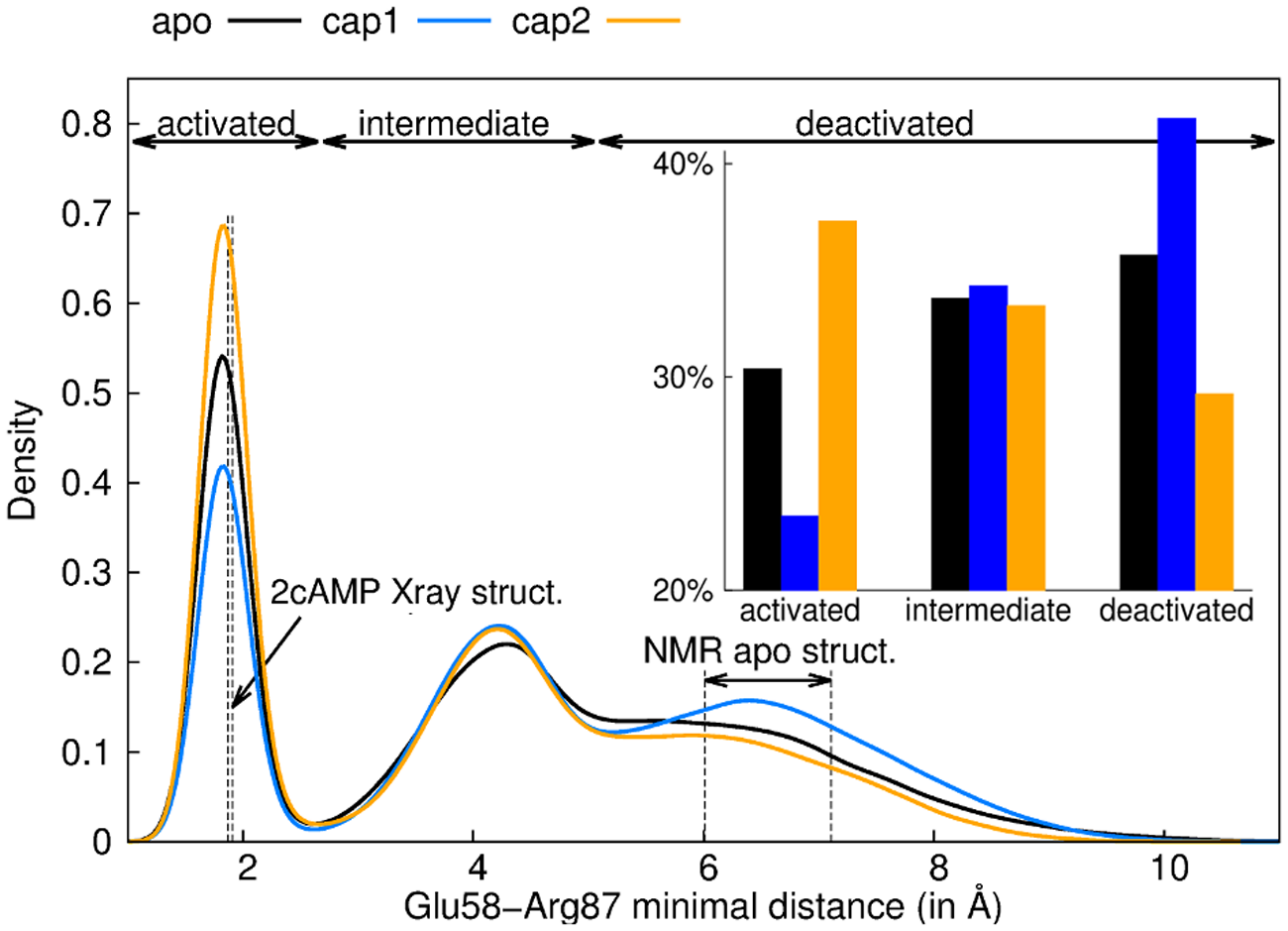
Glu58 and Arg87 minimal distance decreases upon activation. Probability distribution of the minimal distance between Glu68 and Arg87 for apo (black), cap1 (blue) and cap2 (orange) states. Both protomers were taken into account. Vertical dashed lines represent the respective values as observed in the cap2 X-ray structure (1G6N) and the range covered in the apo NMR structures (2WC2 [25]). The integration limits used in the inset for the three states (activated, intermediate and deactivated) have been determined by the two inflection points of the cap2 distribution (2.71 and 5.11 for intermediate and deactivated respectively).

Interestingly, Asp68, which played a key role in the first binding event, is also important after the binding of the second nucleotide. Asp68 propagated the perturbation due to cAMP binding symmetrically to the anti-parallel β-sheet core and then reach the β4/β5-hairpin in close contact with the DBD This symmetric pathway, bridging Asp68 to Glu58, involves sequentially Leu64, Val47, Ser46, Lys89, Ala88 and Arg87. In this network, main chains play an important role. The β-bundle which connect the cAMP pocket with the NBD-DBD interface acts as a good signal propagator through H-bond interactions. We observed in both protomers a significant change in the interaction between Glu58 (β-hairpin) and Arg87 (β-strand 2, Fig 5D) side chains, the last residue pair of this network. Structural data has suggested the distance Glu58-Arg87 to critically change as a function of the activation state [9,25], namely to decrease by about 5 Å upon CAP activation. We observed the same trend in our MD simulations: the Glu58-Arg87 minimal distance was larger in the apo and cap1 states in comparison to the cap2 state, for both protomers (Fig 6). Interestingly, the network further reaches Gln174 located on the DBD in the second protomer. We computed dihedral order parameter difference for each residue from the cap1 and the cap2 simulations (S6 Fig), and obtained significant changes for some of the key residues highlighted by FDA. We observed stiffening of Glu77’, Glu78’, Gly79’ and Gln80’, due to their enhanced interaction with Arg122.

## Discussion

We here aimed at deciphering the allosteric mechanism of CAP to explain the anticooperativity of cAMP binding and the cAMP-dependent activation of CAP for DNA binding. We performed MD simulations of three CAP states, without cAMP (apo), with a single (cap1) and two bound cAMP molecules (cap2). Experimental NMR data [8] showed that the anticooperativity in CAP is of mainly entropic nature, with changes in atomic fluctuations upon ligand binding around largely unaffected mean positions of the protein coordinates. We were able to semi-quantitatively reproduce the entropic penalty of anticooperative cAMP binding from atomic force correlations using a recently developed force-covariance (FC) entropy estimator [30]. Furthermore, to investigate the mechanism of negative cooperativity and CAP activation, we used FDA to reveal changes in the protein’s internal force network upon cAMP binding. FDA gave insights into the allosteric mechanisms of CAP, which helped to identify minute allosteric rearrangements at the domain interfaces. We obtained allosteric networks for the first and second binding event, which both span the two nucleotide binding pockets, but only for the second cAMP binding also reach into the DBD. Calculated pathways involve the amino acid pairs Glu72’-Arg123’ and Leu73-Leu124’ (pathway A) and Arg122, Glu77’ and Arg123 (pathway B). Using FDA and subsequent structural analysis, we identified critical residues along the signal propagation pathways, the functional role of which are partially supported by mutational studies. For instance, it has been shown that mutating Glu72, a residue we find within an allosteric link to Arg123, impacts cAMP binding and allostery in CAP [32]. Likewise, a mutation of Arg123, which FDA suggests to be implicated in both communication pathways, modifies CAP activity [33].

Our studies additionally revealed Glu58, located at the β4/β5 hairpin, as being involved in force transmission towards the DBD, as this crucial site symmetrically stands out in the force networks of both protomers (Figs 5D and 6). This suggests this residue as an interesting candidate for mutations that we predict to result in decoupling of the allosteric regulation of DNA binding from that of cAMP binding. Similarly, residue pairs involved in the signal transmission toward the DBD (Ser46, Val47, Arg87, Ala88 and Lys89) could be good candidates for mutations in order to decouple cAMP binding from CAP activation. In this network, FDA delineates especially Asp68 to be crucial for global signal transmission in CAP, suggesting it as another interesting previously untested mutagenesis candidate.

Our data complements and allows the interpretation of the enormous collection of insightful X-ray and NMR data on CAP structure, dynamics and allostery. Previous conclusions on CAP allostery have relied on averaged NMR data (chemical shifts or order parameters) from the two CAP protomers justified by assuming symmetry of the CAP dimer. By contrast, we here find this symmetry to hold only partially. In particular, we obtained allosteric pathways between the CAP protomers, crossing the α-helix H3, which break the symmetry. Our results indicate that it would be desirable to address the challenge to distinguish between the protomers of CAP even in the apo or cap2 states, when collecting experimental data. We expect the CAP force networks to differ from networks of correlated fluctuations, as the latter relies on high amplitude motions and thus occur in more flexible regions such as loops [21]. Systematically analysing the different features revealed by correlated coordinate fluctuations as obtained from PCA or driven MD simulations [35] as opposed to those from our force network for a set of allosteric proteins would in this regard be of interest.

While our simulations recover the experimentally observed entropic penalty of the second binding event, we also identified shifts in the mean structure of the protein that can additionally give rise to the anti-cooperativity for cAMP binding. More specifically, significant side chain adjustments right at the cAMP binding pocket and the protomer-protomer interface hamper the binding of the second cAMP. In particular, Arg123’ (Fig 4A), which we proved that it is a key residue for the allosteric signalling in CAP, in the cap1 state populated a region normally occupied by cAMP, thereby occluding the empty binding cleft more than in to apo state. This “enthalpic” component is not in contradiction with NMR experiments, for which minor mean displacements of side-chains, in particular those lacking methyl groups, are challenging to track.

Overall, our work extends the entropy-centric view on CAP. It suggests atomic forces and stresses, which intriguingly have been previously shown to be a signature of folded proteins [36], as a useful measure of protein regulation—with force covariance as an entropy estimator and force distribution as a tool to reveal allosteric communication independently of the nature of the allosteric mechanism, being it structural, dynamic, or both.

## Material and Methods

### Molecular Dynamics Simulations

The MD simulations were performed using GROMACS 4.0.5 [37] with the AMBER force field 03 [38]. The parameters for the cAMP molecule were determined with the Generalized Amber Force Field [39] in conjunction with the program Antechamber [40]. The crystal structure of the E. coli catabolite activator protein (PDB id:1G6N [9], Uniprot id: P0ACJ8) was used for the MD simulations. The protonation states of the amino acids were calculated with the WHAT IF software package [41]. A triclinic simulation box was filled with TIP3P water [42] and sodium/chloride ions at a physiological concentration of 120 mM with a resulting overall system charge of zero. All simulations were run in the NpT ensemble. The temperature was kept constant at 300 K by coupling to the Nose-Hoover thermostat with τt = 0.1 ps. The pressure was kept constant at p = 1 bar using isotropic coupling to a Parrinello-Rahman barostat with τp = 1 ps and a compressibility of 4.5x10^-5^ bar^−1^. After energy minimization, the LINCS algorithm [43] was used to constrain all bonds. Lennard-Jones interactions were calculated using a cut-off of 1 nm. Long-range electrostatics were calculated by Particle-Mesh Ewald (PME) summation [44]. Every state (apo, one cAMP bound—cap1, two cAMP bound—cap2) of the CAP system was minimized, using the steepest descent algorithm. For each state, nine trajectories with different random starting velocities were calculated, first, in a 200 ps position restraint run (restraint force constant = 1000 kJ•mol^−1^.nm^2^; time step = 2 fs), followed by a 6 ns equilibration run and a 100 ns production run (time step = 2 fs), resulting in 27 trajectories with a total length of 2862 ns. Only the 100 ns production runs were further analysed. System coordinates were saved every 20 ps, resulting in 45,000 conformations for each state (apo, cap1 and cap2). The average size of the triclinic simulation box was 89 x 101 x 97 Å, resulting in a system volume of about 87,0000 Å^3^ with about 87,000 atoms.

### Principal Component Analysis and Motion Comparisons

We performed Principal Component Analyses (PCA) on concatenated trajectories of either apo, cap1 or cap2 states. A PCA consists in diagonalizing the co-variance matrix of Cartesian coordinates, in order to delineate collective motions sampled during our MD simulations. Eigenvectors describe motions and eigenvalues inform about the amplitude of the corresponding motions. To reduce the number of degrees of freedom, only the main-chain atoms were taken into account to build the co-variance matrices. We further compared only the first five most collective motions to experimentally known ones, such as the activation or the DNA binding motions. The motion were compared in the 3D space using motions matrices which is described in detail elsewhere [45,46]. A motion matrix is created using a difference of two Cα-Cα distance matrices. We then computed a correlation coefficient between the two motion matrices. The method does not need any fitting as Cα-Cα distance matrices are signatures in internal coordinates.

### Squared Generalized Order Parameters for the Methyl Group Symmetry Axis

In order to validate our simulations, we have computed squared generalized order parameters for the Methyl Group Symmetry Axis (S^2^_axis_). S^2^_axis_ were computed for all terminal C-CH3 bond vectors (or S-CH3 in the case of methionines) using multiple windows of 3 ns, as described by Hu et al. [22]. A S^2^axis C-CH3 bond vector in the 3D space x, y and z, is defined as follow:

$$S_{axis}^{2}=\frac{3}{2}\left[{\langle x^{2}\rangle }^{2}+{\langle y^{2}\rangle }^{2}+{\langle z^{2}\rangle }^{2}+2{\langle xy^{2}\rangle }^{2}+{\langle xz^{2}\rangle }^{2}+{\langle yz^{2}\rangle }^{2}\right]-\frac{1}{2}$$ (1)

### Dihedral Order Parameters

To have a broad view of the CAP dynamics, and not only dynamical behaviour residues which possess a methyl group, we computed dihedral order parameters for every residues of the protein, as implemented in GROMACS 4.5.3 [47] and described by Van der Spoel and Berendsen [48]. This analysis was done on concatenated trajectories of the three states (apo, cap1 and cap2). We then computed order parameters differences residue per residue, to determine dynamical behaviour modification of amino acids after the two cAMP binding events.

### Force Distribution Analysis (FDA)

In the Force Distribution Analyses (FDA) [15,16], the forces between each atom pair i and j were analysed at each trajectory step. All terms in the force field were considered, including both non-bonded and bonded terms, except forces including water and ions, as well as PME forces. The more recent FDA version [16] in conjunction with GROMACS 4.5.3 [47] was used here. For a residue-wise analysis, inter-residue forces *F*_*uv*_ were calculated from the norm (magnitude) of the force vector resulting from summing up over all force vectors F_ij_ between atom pairs *i* and *j* within the two residues *u* and *v*:

$$F_{uv}=\left\|\sum_{ij}{\vec{F}}_{ij}\right\|;i\in u,j\in v$$ (2)

We note that time averaged pairwise forces can be different from zero even at equilibrium, in contrast to atomic forces which average to zero over time. To enhance the signal to noise ratio, the pairwise forces *F*_*uv*_ calculated from each frame in the trajectories were averaged over time and over the 9 independent runs of the 3 states, apo, cap1 and cap2. We then computed the changes in time-averaged residue pairwise forces between cap1 and apo as well as cap2 and cap1, to track the signal propagation due to the first and the second cAMP-binding event, respectively. The networks shown in Figs 3 and 5 are connected graphs of force differences beyond a given threshold, which further reduced the noise [20]. The stress *S*, also known as punctual stress, for a residue *u* was here used to monitor convergence of our simulations (S3 Fig). The stress is defined as the sum of all residue pairs F_uv_ acting on the residue *u*:

$$S_{u}=\sum_{v}\left\|{\vec{F}}_{uv}\right\|$$ (3)

## Supporting Information

S1 Fig Internal fluctuation of cap2. Comparison of Root Mean Square Fluctuations (RMSF) during cap2 MD simulations (A) and crystallographic B-factors of the cap2 crystal structure (B, pdb code: 1G6N). (C) Experimental Cα B-factors against Cα B-factors calculated from MD-derived RMSF values for each residue.
(TIFF)

S2 Fig Comparison of squared generalized order parameters for the Methyl Group Symmetry Axis (S^2^axis) observed experimentally to those computed from cap2 simulation. (A) S^2^axis of cap2 simulations (black) and experiment (red) as a function of the C-CH3 bond vector index. The indices reflect their order of appearance in the protein sequence. (B) S^2^axis from our simulations as a function of S^2^axis from experiment for each C-CH3 bond vectors. Linear fitting gives a slope of 0.45 and an offset of 0.27.
(TIFF)

S3 Fig Convergence of pairwise force of cap2 by permutation tests. Mean difference of the punctual stress between the two protomers are shown as a function of the quantity of simulations taken into account. E.g. for the quantity of 3 simulations, the mean difference between the protomer stresses for all combinations of three of the nine cap2 simulations has been used.
(TIFF)

S4 Fig Structural features of key residues pairs highlighted by FDA. (A) Distribution of the non-bonded energy terms (Lennard-Jones and Coulomb) between Arg122 and Glu77’. (B) Hydrogen bond occurrence between Glu77’ and Gln80’.
(TIFF)

S5 Fig Residual dihedral order parameter differences upon binding of the first cAMP. A positive difference means a flexibility gain after binding whereas a negative difference means a flexibility loss. Amino acids described in the text are labelled.
(TIFF)

S6 Fig Residual dihedral order parameter differences upon binding of the second cAMP. A positive difference means a flexibility gain after binding whereas a negative difference means a flexibility loss. Amino acids described in the text are labelled.
(TIFF)

S1 Movie Inactivation motion followed by the apo state. Superimposition of the motion described by the 4th eigenvector of the apo state (cyan) and the two experimentally known structures (active in
(GIF)
