# Supplementary figures and images for: Dynamic Allostery of the Catabolite Activator Protein Revealed by Interatomic Forces
Source: PLoS Comput Biol. 2015 Aug 5;11(8):e1004358. doi: 10.1371/journal.pcbi.1004358 (PMC4526232; doi:10.1371/journal.pcbi.1004358)

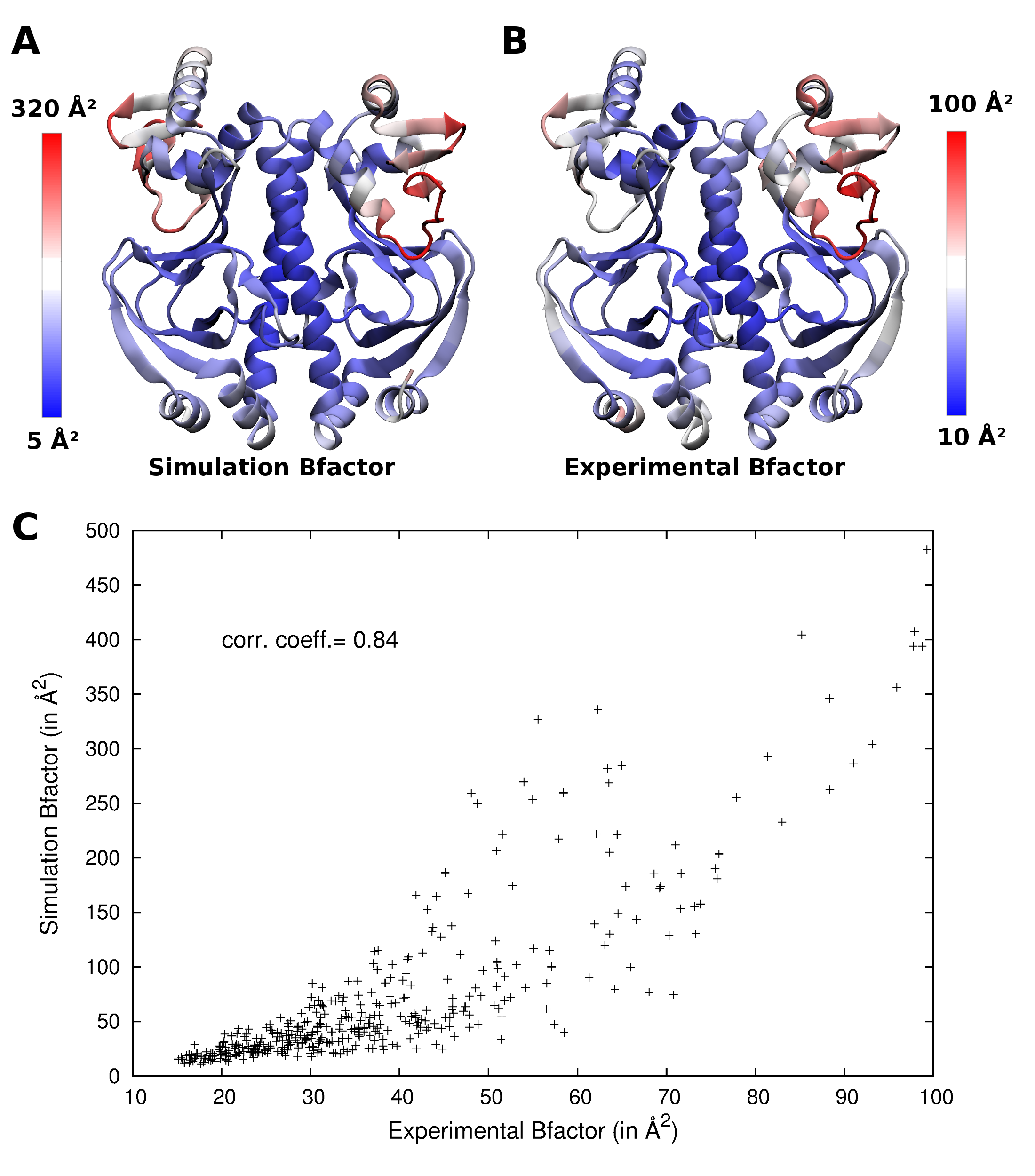

Supplement: S1 Fig — Comparison of Root Mean Square Fluctuations (RMSF) during cap2 MD simulations (A) and crystallographic B-factors of the cap2 crystal structure (B, pdb code: 1G6N). (C) Experimental Cα B-factors against Cα B-factors calculated from MD-derived RMSF values for each residue. (TIFF) [file pcbi.1004358.s001.tiff]

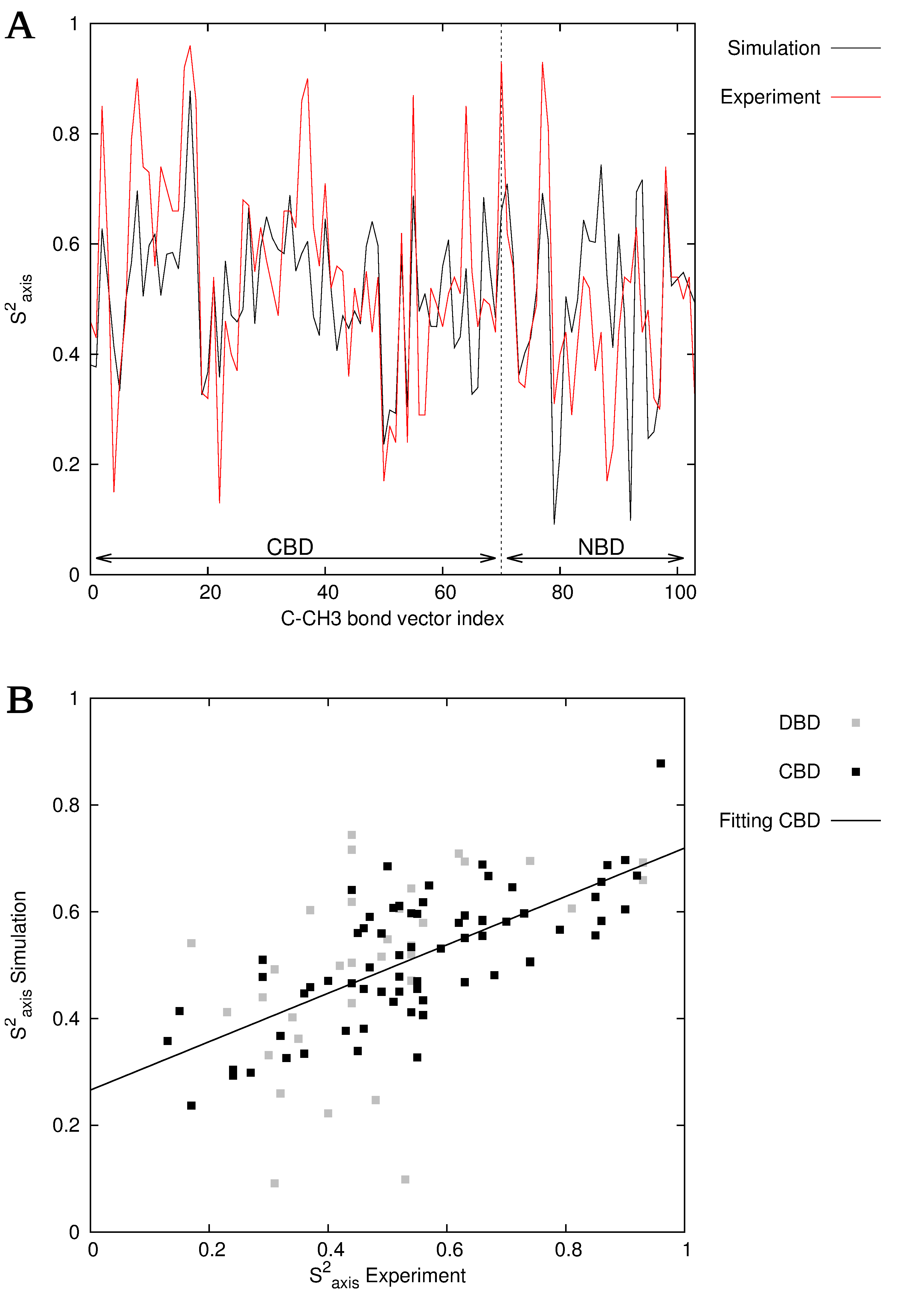

Supplement: S2 Fig — (A) S2axis of cap2 simulations (black) and experiment (red) as a function of the C-CH3 bond vector index. The indices reflect their order of appearance in the protein sequence. (B) S2axis from our simulations as a function of S2axis from experiment for each C-CH3 bond vectors. Linear fitting gives a slope of 0.45 and an offset of 0.27. (TIFF) [file pcbi.1004358.s002.tiff]

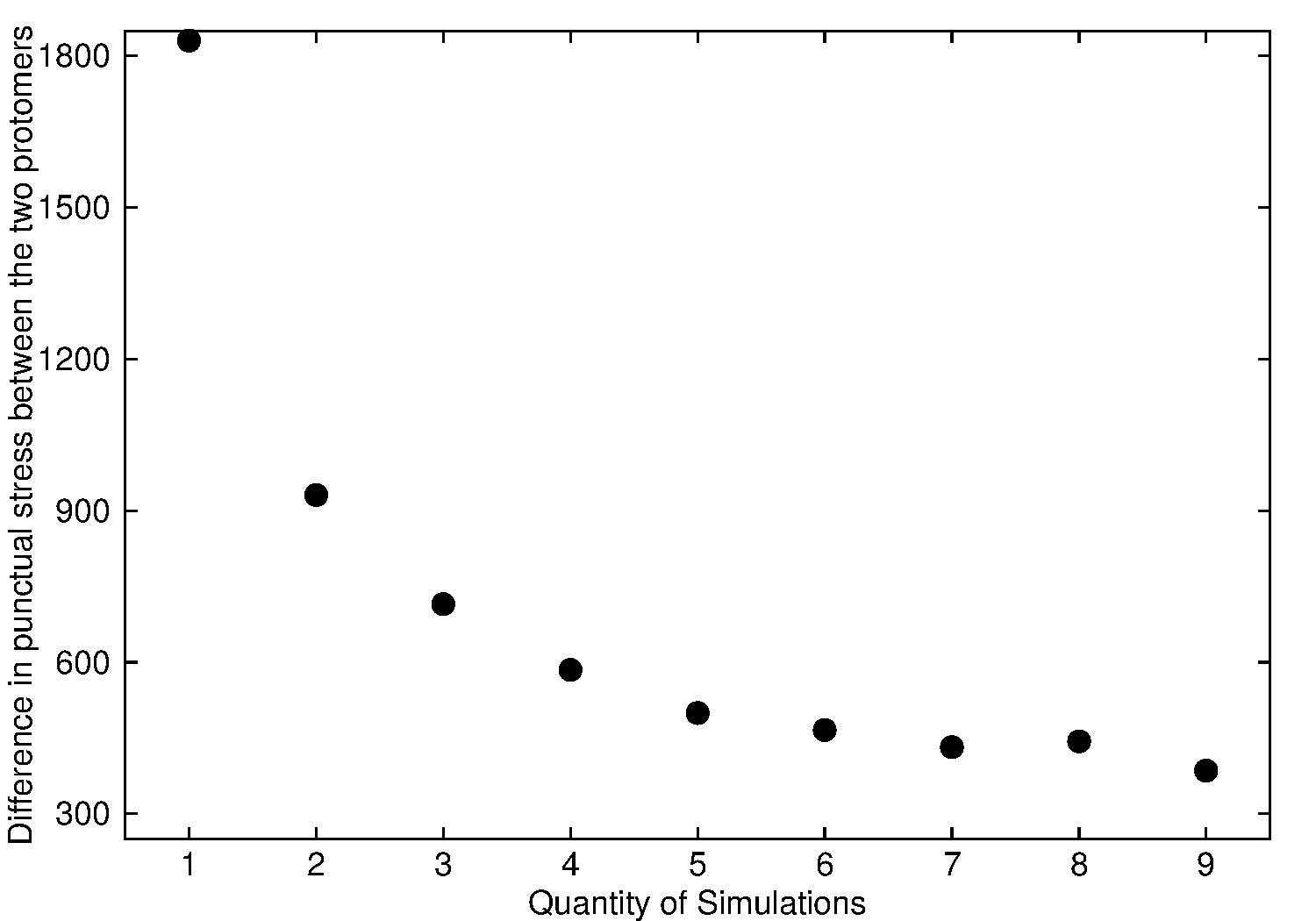

Supplement: S3 Fig — Mean difference of the punctual stress between the two protomers are shown as a function of the quantity of simulations taken into account. E.g. for the quantity of 3 simulations, the mean difference between the protomer stresses for all combinations of three of the nine cap2 simulations has been used. (TIFF) [file pcbi.1004358.s003.tiff]

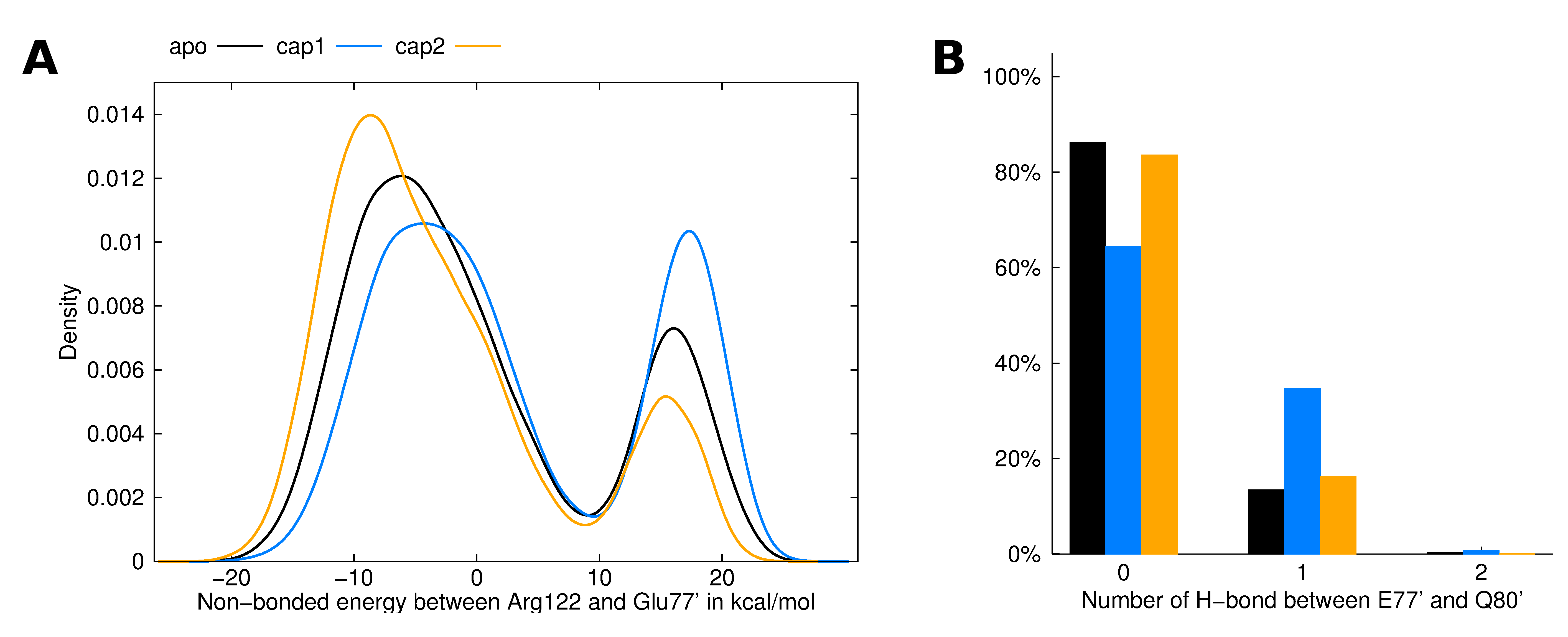

Supplement: S4 Fig — (A) Distribution of the non-bonded energy terms (Lennard-Jones and Coulomb) between Arg122 and Glu77’. (B) Hydrogen bond occurrence between Glu77’ and Gln80’. (TIFF) [file pcbi.1004358.s004.tiff]

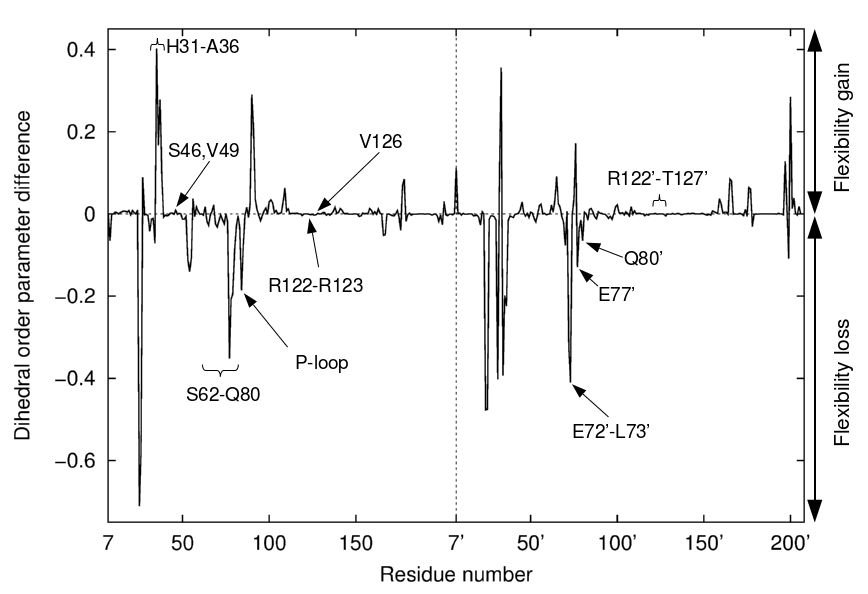

Supplement: S5 Fig — A positive difference means a flexibility gain after binding whereas a negative difference means a flexibility loss. Amino acids described in the text are labelled. (TIFF) [file pcbi.1004358.s005.tiff]

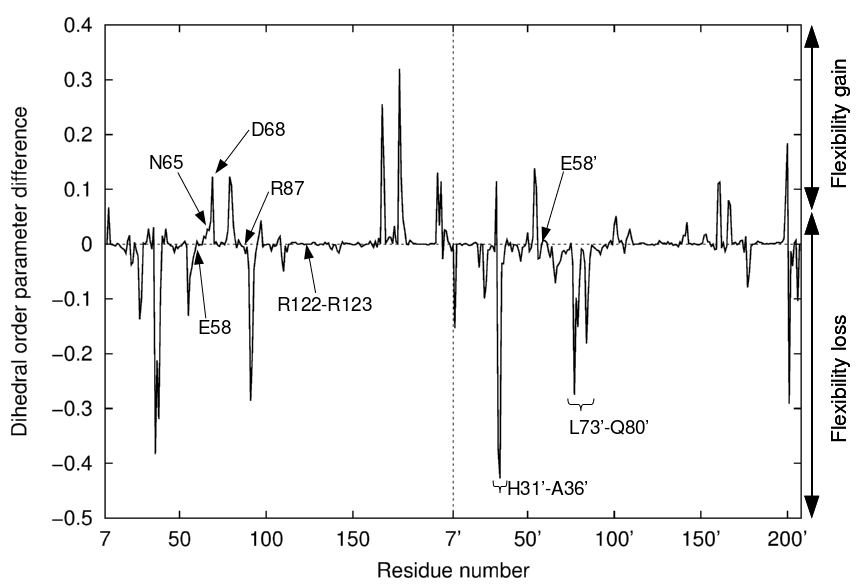

Supplement: S6 Fig — A positive difference means a flexibility gain after binding whereas a negative difference means a flexibility loss. Amino acids described in the text are labelled. (TIFF) [file pcbi.1004358.s006.tiff]

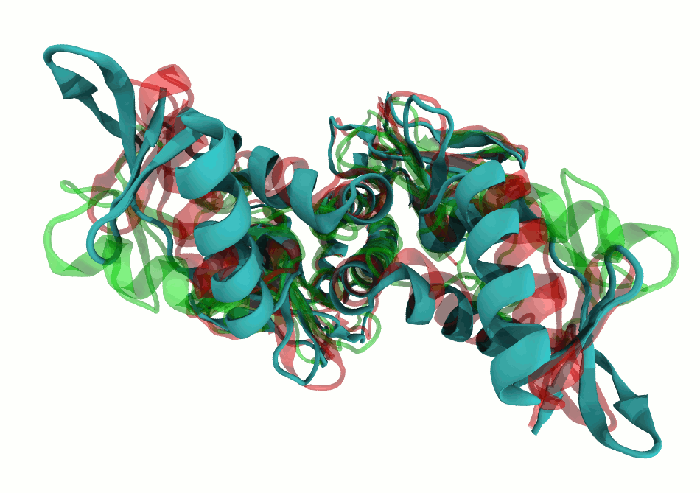

Supplement: S1 Movie — Superimposition of the motion described by the 4th eigenvector of the apo state (cyan) and the two experimentally known structures (active in (GIF) [file pcbi.1004358.s007.gif]
